# Elucidating the genetic architecture of Adams–Oliver syndrome in a large European cohort

**DOI:** 10.1002/humu.23567

**Published:** 2018-07-04

**Authors:** Josephina A.N. Meester, Maja Sukalo, Kim C. Schröder, Denny Schanze, Gareth Baynam, Guntram Borck, Nuria C. Bramswig, Duygu Duman, Brigitte Gilbert‐Dussardier, Muriel Holder‐Espinasse, Peter Itin, Diana S. Johnson, Shelagh Joss, Hannele Koillinen, Fiona McKenzie, Jenny Morton, Heike Nelle, Willie Reardon, Claudia Roll, Mustafa A. Salih, Ravi Savarirayan, Ingrid Scurr, Miranda Splitt, Elizabeth Thompson, Hannah Titheradge, Colm P. Travers, Lionel Van Maldergem, Margo Whiteford, Dagmar Wieczorek, Geert Vandeweyer, Richard Trembath, Lut Van Laer, Bart L. Loeys, Martin Zenker, Laura Southgate, Wim Wuyts

**Affiliations:** ^1^ Center of Medical Genetics University of Antwerp and Antwerp University Hospital Antwerp Belgium; ^2^ Institute of Human Genetics University Hospital Magdeburg Magdeburg Germany; ^3^ Genetic Services of Western Australia and the Western Australian Register of Developmental Anomalies King Edward Memorial Hospital Perth Australia; ^4^ Telethon Kids Institute Perth Australia; ^5^ School of Paediatrics and Child Health University of Western Australia Perth Australia; ^6^ Institute of Human Genetics University of Ulm Ulm Germany; ^7^ Institut für Humangenetik Universitätsklinikum Essen, Universität Duisburg‐Essen Essen Germany; ^8^ Division of Pediatric Genetics Ankara University School of Medicine Ankara Turkey; ^9^ Service de Génétique, CHU de Poitiers University of Poitiers Poitiers France; ^10^ Guy's Regional Genetics Service Guy's and St Thomas' NHS Foundation Trust London United Kingdom; ^11^ Department of Dermatology Basel University Hospital Basel Switzerland; ^12^ Department of Clinical Genetics Sheffield Children's NHS Foundation Trust Sheffield United Kingdom; ^13^ West of Scotland Clinical Genetics Service Queen Elizabeth University Hospital Glasgow United Kingdom; ^14^ Department of Clinical Genetics Helsinki University Hospital Helsinki Finland; ^15^ Genetic Services of Western Australia King Edward Memorial Hospital for Women Subiaco Australia; ^16^ West Midlands Regional Clinical Genetics Service and Birmingham Health Partners Birmingham Women's Hospital NHS Foundation Trust Birmingham United Kingdom; ^17^ MVZ für Pränatalmedizin und Genetik Nürnberg Germany; ^18^ Clinical Genetics National Maternity Hospital Dublin Ireland; ^19^ Abteilung Neonatologie und Pädiatrische Intensivmedizin, Vestische Kinder‐ und Jugendklinik Datteln Universität Witten/Herdecke Datteln Germany; ^20^ Division of Pediatric Neurology, Department of Pediatrics King Khalid University Hospital and College of Medicine, King Saud University Riyadh Saudi Arabia; ^21^ Victorian Clinical Genetics Services, Murdoch Children's Research Institute and the University of Melbourne Melbourne Australia; ^22^ Bristol Genetics Service, University Hospitals Bristol NHS Foundation Trust St Michael's Hospital Bristol United Kingdom; ^23^ Northern Genetics Service Newcastle upon Tyne Hospitals NHS Foundation Trust Newcastle upon Tyne United Kingdom; ^24^ South Australian Clinical Genetics Service, North Adelaide, South Australia, Australia SA Clinical Genetics Service, SA Pathology at the Women's and Children's Hospital North Adelaide SA Australia; ^25^ School of Medicine University of Adelaide North Terrace Adelaide SA Australia; ^26^ Division of Neonatology University of Alabama at Birmingham Birmingham USA; ^27^ Centre de Génétique Humaine Université de Franche‐Comté Besançon France; ^28^ West of Scotland Genetic Services Queen Elizabeth University Hospital Glasgow United Kingdom; ^29^ Institute of Human Genetics, Medical Faculty Heinrich Heine University Düsseldorf Germany; ^30^ Division of Genetics & Molecular Medicine, King's College London Faculty of Life Sciences & Medicine, Guy's Hospital London United Kingdom; ^31^ Molecular and Clinical Sciences Research Institute St George's University of London London United Kingdom

**Keywords:** Adams–Oliver syndrome, genetics, mutation screening, Notch signaling, Rho GTPase

## Abstract

Adams–Oliver syndrome (AOS) is a rare developmental disorder, characterized by scalp aplasia cutis congenita (ACC) and transverse terminal limb defects (TTLD). Autosomal dominant forms of AOS are linked to mutations in *ARHGAP31*, *DLL4*, *NOTCH1* or *RBPJ*, while *DOCK6* and *EOGT* underlie autosomal recessive inheritance. Data on the frequency and distribution of mutations in large cohorts are currently limited. The purpose of this study was therefore to comprehensively examine the genetic architecture of AOS in an extensive cohort. Molecular diagnostic screening of 194 AOS/ACC/TTLD probands/families was conducted using next‐generation and/or capillary sequencing analyses. In total, we identified 63 (likely) pathogenic mutations, comprising 56 distinct and 22 novel mutations, providing a molecular diagnosis in 30% of patients. Taken together with previous reports, these findings bring the total number of reported disease variants to 63, with a diagnostic yield of 36% in familial cases. *NOTCH1* is the major contributor, underlying 10% of AOS/ACC/TTLD cases, with *DLL4* (6%), *DOCK6* (6%), *ARHGAP31* (3%), *EOGT* (3%), and *RBPJ* (2%) representing additional causality in this cohort. We confirm the relevance of genetic screening across the AOS/ACC/TTLD spectrum, highlighting preliminary but important genotype–phenotype correlations. This cohort offers potential for further gene identification to address missing heritability.

## 1 INTRODUCTION

Adams–Oliver syndrome (AOS) is a rare developmental disorder characterized by both aplasia cutis congenita (ACC) of the scalp and transverse terminal limb defects (TTLD), including hypoplastic nails, brachydactyly, oligodactyly, and amputation defects (Adams & Oliver, 1945; Snape et al., 2009). Additional abnormalities affect the cardiovascular and neurological systems. Approximately 20% of AOS patients have congenital heart defects, including atrial septal defect (ASD), ventricular septal defect (VSD), tetralogy of Fallot (TOF), as well as valvular and ventricular abnormalities. A similar proportion is affected by vascular anomalies, for example, cutis marmorata telangiectatica congenita (CMTC). Neurological abnormalities occur less frequently and include intracranial abnormalities (e.g., calcification, cortical dysplasia, and gliosis), developmental delay, intellectual disability, epilepsy, and microcephaly (Digilio, Marino, Baban, & Dallapiccola, 2015; Lehman, Wuyts, & Patel, 2016; Snape et al., 2009).

Multiple causative genes have been discovered for AOS over the past few years. Heterozygous mutations in *ARHGAP31* (MIM# 100300), *RBPJ* (MIM# 614814), *NOTCH1* (MIM# 616028), or *DLL4* (MIM# 616589) have been described in autosomal dominant and sporadic cases, while autosomal recessive forms of AOS may be due to biallelic mutations in *DOCK6* (MIM# 614219) or *EOGT* (MIM# 615297) (Cohen et al., 2014; Hassed et al., 2012; Lehman et al., 2014; Meester et al., 2015; Shaheen et al., 2011, 2013; Southgate et al., 2011, 2015; Stittrich et al., 2014; Sukalo et al., 2015). The NOTCH pathway plays a major role in AOS pathogenesis, with four causal genes (*RBPJ*, *NOTCH1*, *DLL4*, and *EOGT*) involved in Notch signaling. Specifically, DLL4 is a ligand of the Notch receptors (NOTCH1‐4), while RBPJ is the major transcriptional regulator for Notch signaling, modulated by its transcriptional complex with the Notch intracellular domain, which is cleaved upon activation of the pathway (Bray, 2006). EOGT is an epidermal growth factor (EGF) domain‐specific O‐linked *N*‐acetylglucosamine transferase and, although its function remains relatively poorly characterized in humans, it has been shown to act on EGF domain–containing proteins, including the Notch receptors in mammals (Sakaidani et al., 2012). By contrast, *ARHGAP31* and *DOCK6* are not directly linked to Notch signaling, but instead encode regulatory proteins that specifically control the activity of the Rho GTPases RAC1 and CDC42, which are important for the maintenance of the actin cytoskeleton (Southgate et al., 2011).

AOS has an estimated frequency of one affected individual per 225,000 live births (Martinez‐Frias et al., 1996). Due to the rarity of this disorder and relatively recent identification of causal genes, the percentage of AOS cases attributable to each of the established AOS genes in large cohorts remains unclear. Importantly, gaining a better understanding of potential genotype–phenotype correlations in this condition may identify “at‐risk” individuals who have an increased likelihood of developing additional medical complications. Here, we report on the molecular characterization of an extensive cohort of AOS/ACC/TTLD probands and their family members, providing further clarity with regard to the interpretation of identified variants and potential for improved molecular diagnosis and clinical management of these patients.

## 2 MATERIALS AND METHODS

### 2.1 Patient cohort

All patients and families were recruited through the European AOS Consortium, and all participants provided informed written consent to participate in the study. The study was approved by the appropriate institutional ethics review boards. Patients were diagnosed according to the diagnostic criteria proposed by Snape et al. (2009). Specifically, the presence of two major criteria (TTLD, ACC, or a documented family history) or one major and one minor feature (CMTC, congenital cardiac defect, or vascular anomaly) was considered strongly indicative of AOS. Patients with ACC or TTLD in the absence of any associated family history of AOS or other syndromic features were classified as isolated ACC or isolated TTLD, respectively. Based on the diagnostic criteria proposed by Lehman et al. (2016), the presence of a (likely) pathogenic variant in an autosomal dominant AOS‐related gene or a biallelic (likely) pathogenic variant in an autosomal recessive AOS‐related gene was also considered a major criterion. A total of 194 families/probands were included in this study; only the proband for each family was used for the calculation of frequencies, yields, and counts. All affected individuals of the family were taken into consideration for description of the clinical features.

### 2.2 Sequencing

All AOS/ACC/TTLD patients were screened for mutations in the six established genes (*ARHGAP31*: NM_020754.3; *DLL4*: NM_019074.3; *DOCK6*: NM_020812.3; *EOGT*: NM_001278689.1; *NOTCH1*: NM_017617.4; *RBPJ*: NM_005349.3). The majority of the samples were sequenced by targeted next‐generation resequencing (*n* = 140) using either the HaloPlex Target Enrichment System (Agilent Technologies, Santa Clara, CA) as described previously (Meester et al., 2015), or a TruSeq Custom Amplicon Panel (Illumina, San Diego, CA) followed by sequencing on a MiSeq system (Illumina, San Diego, CA) with 150 bp paired‐end reads. Sequence data obtained from the TruSeq Custom Amplicon Panel were analyzed using Illumina's VariantStudio Data Analysis Software v3.0. GRCh37 was used as the reference human genome build. The remaining patients were screened by either whole‐exome sequencing (WES, *n* = 28) or Sanger sequencing (*n* = 26) as previously described (Southgate et al., 2015; Sukalo et al., 2015). ANNOVAR (Wang, Li, & Hakonarson, 2010) dbNSFPv3.0.a (Liu, Jian, & Boerwinkle, 2011) annotation was used for *in silico* prediction scores, including MutationTaster (Schwarz, Cooper, Schuelke, & Seelow, 2014), SIFT (Kumar, Henikoff, & Ng, 2009), PolyPhen2 hvar (Adzhubei et al., 2010), and CADD (Supporting Information Table S1) (Kircher et al., 2014). Alamut (v2.8.1) was used for *in silico* splicing predictions, including SpliceSiteFinder‐like, MaxEntScan, NNSPLICE, GeneSplicer, and Human Splicing Finder (Supporting Information Table S2). After identification of a likely pathogenic variant by Sanger sequencing of single genes, no further screening of the remaining AOS genes was performed. All observed mutations were confirmed by conventional Sanger sequencing on an independent sample.

### 2.3 Variant classification

Variants are classified according to the American College of Medical Genetics (ACMG) guidelines (Richards et al., 2015). However, we have used a few additional gene‐specific criteria, in consideration of the pathogenic mechanisms involved in AOS. First, all protein‐truncating mutations in the last exon of *ARGHAP31* were classified as pathogenic due to gain‐of‐function, in accordance with the previously reported mechanism in this gene (Southgate et al., 2011). Second, cysteine substitutions within EGF domains of *DLL4* or *NOTCH1* were considered to have strong evidence of pathogenicity, similar to null variants (Dietz, Saraiva, Pyeritz, Cutting, & Francomano, 1992; Schrijver, Liu, Brenn, Furthmayr, & Francke, 1999). Third, recurrent missense mutations affecting the same amino acid in independent cases were classified as pathogenic due to multiple occurrences. Lastly, in families where ≥3 individuals were available for screening, any variant with a penetrance less than 60% was classified as a variant of uncertain significance (VUS).

## 3 RESULTS

**Figure 1:**
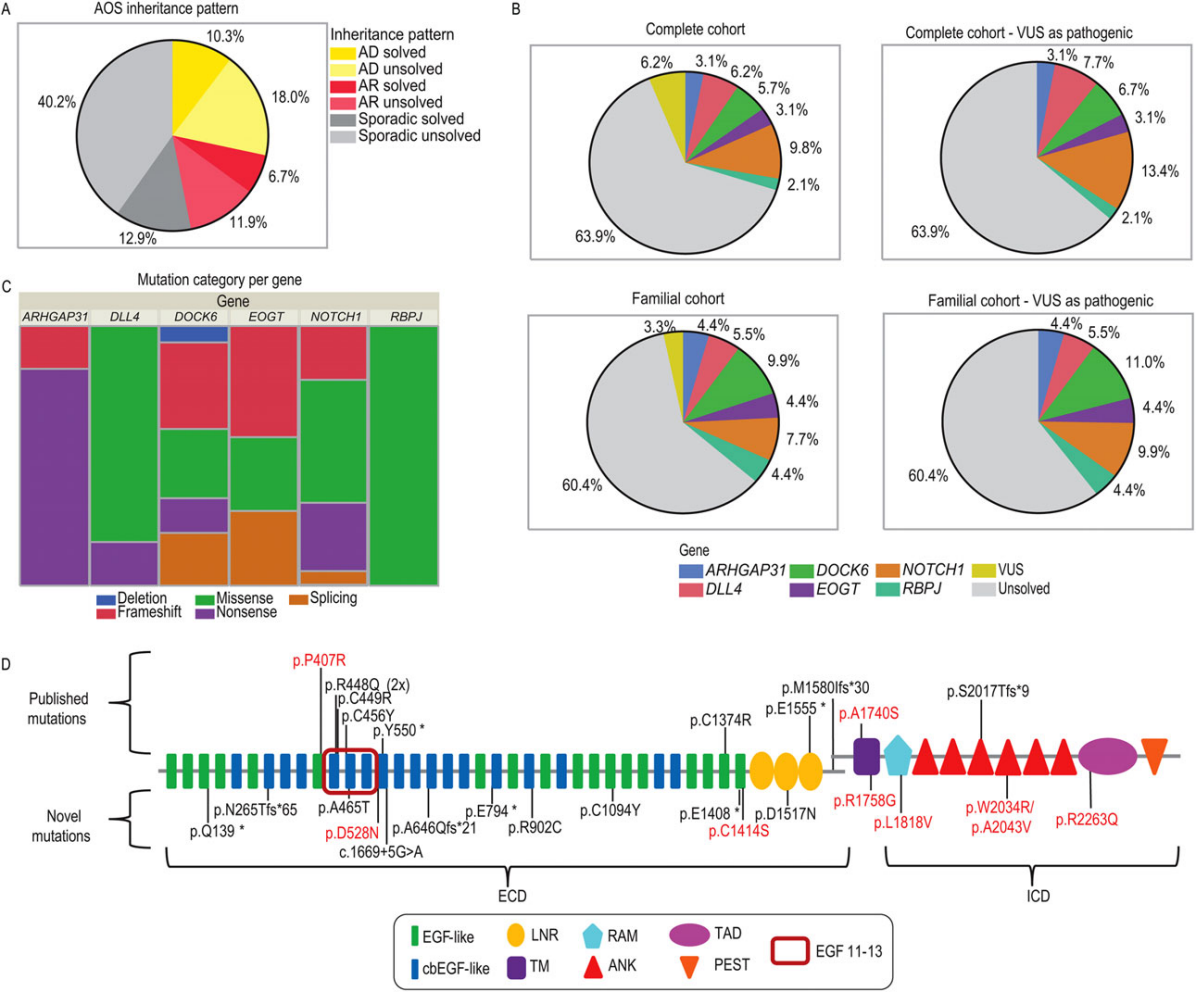
Genetic architecture of AOS. (**a**) Distribution of inheritance pattern across our AOS cohort. AD, autosomal dominant; AR, autosomal recessive. (**b**) Contribution of mutations in each of the six known genes to the development of AOS in the complete cohort and in familial cases only. For each cohort, charts depict the gene distribution excluding VUS and when all VUS are considered as pathogenic, respectively. (**c**) Distribution of mutation categories across established AOS genes. (**d**) Representation of previously reported and novel pathogenic mutations in NOTCH1. Mutations identified in this study that were previously reported in AOS patients are represented on the upper part of the figure. Novel mutations are arrayed below the schematic. EGF 11–13 highlights the ligand‐binding domain. Pathogenic mutations are depicted in black and VUS in red. ECD, Extracellular domain; ICD, Intracellular domain; EGF‐like, epidermal growth factor‐like domain; cbEGF‐like, calcium‐binding epidermal growth factor‐like domain; LNR, Lin‐12/Notch repeat; TM, transmembrane domain; RAM, RBP‐Jkappa‐associated module; ANK, ankyrin; TAD, transcriptional activation domain and PEST, proline (P), glutamic acid (E), serine (S), and threonine (T)‐rich peptide sequence.

The analyzed cohort comprised 194 distinct AOS/ACC/TTLD familial or sporadic cases. Of these, 36 families were consistent with an autosomal recessive mode of inheritance, based on pedigree data or known consanguinity, while autosomal dominant inheritance was the most likely inheritance pattern in 55 families (Figure 1A). The remaining 103 probands were categorized as sporadic in the absence of any family history or known consanguinity. We provide a causal molecular explanation for the phenotype in 58/194 (30%) of AOS/ACC/TTLD probands (Table 1). Among the 63 pathogenic (or likely pathogenic) mutations in this study, 56 were distinct, or nonrecurrent, mutations and 22 mutations have not been reported to date (Supporting Information Figure S1). In addition, we identified several VUS (*n* = 14, Supporting Information Table S3 and Supporting Information Figure S1). The data on novel variants have now been made available in the ClinVar database (https://www.ncbi.nlm.nih.gov/clinvar).

**Table 1:** Identified pathogenic mutations in six established AOS genes

Fam ID	Gene	Zygosity	Nucleotide change^a^	Amino acid change	Type of mutation	Pathogenicity class	Mutation previously reported	Reference of family^b^
*Pedigrees suggestive of autosomal recessive inheritance*:								
4	*DOCK6*	Homozygous	c.484G > T	p.Glu162*	Nonsense	Pathogenic	c	Sukalo et al., 2015 (Family 8)
6	*DOCK6*	Homozygous	c.1296_1297delinsT	p.Gln434Argfs*21	Frameshift	Pathogenic	c	Sukalo et al., 2015 (Family 3)
8	*DOCK6*	Homozygous	c.2520dupT	p.Arg841Serfs*6	Frameshift	Pathogenic	c, d	Sukalo et al., 2015 (Family 10)
9	*DOCK6*	Homozygous	c.3047T > C	p.Leu1016Pro	Missense	Likely pathogenic	c	Sukalo et al., 2015 (Family 1)
10	*DOCK6*	Homozygous	c.3154G > A	p.Glu1052Lys	Missense	Likely pathogenic	c	Sukalo et al., 2015 (Family 5); Prothero et al., 2007
11	*DOCK6*	Homozygous	c.4786C > T	p.Arg1596Trp	Missense	Likely pathogenic	c	Sukalo et al., 2015 (Family 4)
13	*DOCK6*	Homozygous	c.5235+205_6102‐15delinsCATGGGGCTG	4.3 kb deletion	Deletion	Pathogenic	c	Sukalo et al., 2015 (Family 9)
5	*DOCK6*	Compound heterozygous	c.788T > A	p.Val263Asp	Missense	Likely pathogenic	c	Sukalo et al., 2015 (Family 6); Orstavik et al., 1995
			c.5939+2T > C	p.?	Splicing	Pathogenic		
7	*DOCK6*	Compound heterozygous	c.1902_1905delGTTC	p.Phe635Profs*32	Frameshift	Pathogenic	c	Sukalo et al., 2015 (Family 7)
			c.4106+5G > T	p.?	Splicing	Likely pathogenic		
1	*EOGT*	Homozygous	c.311+1G > T	p.?	Splicing	Pathogenic	Novel mutation	‐
2	*EOGT*	Homozygous	c.404G > A	p.Cys135Tyr	Missense	Pathogenic	Novel mutation	‐
3	*EOGT*	Homozygous	c.1130G > A	p.Arg377Gln	Missense	Likely pathogenic	d	Temtamy et al., 2007 (Family 2)
59	*EOGT*	Compound heterozygous	c.78_81delTCAC	p.His27Alafs*46	Frameshift	Pathogenic	Novel mutation	Verdyck et al., 2003 (Family 4)
			c.1335‐1G > A	p.?	Splicing	Pathogenic	Novel mutation	
*Pedigrees suggestive of autosomal dominant inheritance*:								
40	*ARHGAP31*	Heterozygous	c.2047C > T	p.Gln683*	Nonsense	Pathogenic	c	Southgate et al., 2011 (Family AOS‐12); Bonafede & Beighton, 1979
41	*ARHGAP31*	Heterozygous	c.2047C > T	p.Gln683*	Nonsense	Pathogenic	d	*‐*
42	*ARHGAP31*	Heterozygous	c.2063_2064insTT	p.Ser689*	Nonsense	Pathogenic	c	Isrie et al., 2014
43	*ARHGAP31*	Heterozygous	c.3260delA	p.Lys1087Serfs*4	Frameshift	Pathogenic	c	Southgate et al., 2011 (Family AOS‐5); Verdyck et al., 2006
55	*DLL4*	Heterozygous	c.556C > T	p.Arg186Cys	Missense	Likely pathogenic	c	Meester et al., 2015 (Family 6)
56	*DLL4*	Heterozygous	c.1168T > C	p.Cys390Arg	Missense	Pathogenic	c	Meester et al., 2015 (Family 5)
57	*DLL4*	Heterozygous	c.1365C > G	p.Cys455Trp	Missense	Pathogenic	c	Meester et al., 2015 (Family 3)
58	*DLL4*	Heterozygous	c.1660C > T	p.Gln554*	Nonsense	Pathogenic	c	Meester et al., 2015 (Family 1)
181	*DLL4*	Heterozygous	c.1825C > T	p.Gln609*	Nonsense	Pathogenic	Novel mutation; *de novo*	‐
14	*NOTCH1*	Heterozygous	c.415C > T	p.Gln139*	Nonsense	Pathogenic	Novel mutation	‐
46	*NOTCH1*	Heterozygous	c.794_797delinsCC	p.Asn265Thrfs*65	Frameshift	Pathogenic	Novel mutation	‐
47	*NOTCH1*	Heterozygous	c.1649dupA	p.Tyr550*	Nonsense	Pathogenic	c	Southgate et al., 2015 (Family 1), Verdyck et al., 2003 (Family 2)
48	*NOTCH1*	Heterozygous	c.1935_1936delTG	p.Ala646Glnfs*21	Frameshift	Pathogenic	Novel mutation	Savarirayan et al., 1999
50	*NOTCH1*	Heterozygous	c.4120T > C	p.Cys1374Arg	Missense	Pathogenic	c	Southgate et al., 2015 (Family 3)
52	*NOTCH1*	Heterozygous	c.4663G > T	p.Glu1555*	Nonsense	Pathogenic	c	Southgate et al., 2015 (Family 4)
54	*NOTCH1*	Heterozygous	c.6049_6050delTC	p.Ser2017Thrfs*9	Frameshift	Pathogenic	c	Southgate et al., 2015 (Family 2); Dallapiccola et al. 1992 (Patient 2)
44	*RBPJ*	Heterozygous	c.193A > G	p.Arg65Gly	Missense	Likely pathogenic	Novel mutation	*‐*
175	*RBPJ*	Heterozygous	c.196T > G	p.Phe66Val	Missense	Likely pathogenic	Novel mutation	‐
45	*RBPJ*	Heterozygous	c.505A > G	p.Lys169Glu	Missense	Pathogenic	d	‐
178	*RBPJ*	Heterozygous	c.996C > A	p.Ser332Arg	Missense	Likely pathogenic	Novel mutation	‐
*Sporadic probands*:								
17	*ARHGAP31*	Heterozygous	c.2047C > T	p.Gln683*	Nonsense	Pathogenic	d	*‐*
18	*ARHGAP31*	Heterozygous	c.2182C > T	p.Gln728*	Nonsense	Pathogenic	Novel mutation	*‐*
30	*DLL4*	Heterozygous	c.361G > C	p.Ala121Pro	Missense	Likely pathogenic	c; *de novo*	Meester et al., 2015 (Family 8)
31	*DLL4*	Heterozygous	c.572G > A	p.Arg191His	Missense	Likely pathogenic	d	‐
32	*DLL4*	Heterozygous	c.583T > C	p.Phe195Leu	Missense	Likely pathogenic	c	Meester et al., 2015 (Family 9)
182	*DLL4*	Heterozygous	c.949A > C	p.Thr317Pro	Missense	Likely pathogenic	Novel mutation; *de novo*	‐
34	*DLL4*	Heterozygous	c.1169G > A	p.Cys390Tyr	Missense	Pathogenic	c	Meester et al., 2015 (Family 4)
196	*DLL4*	Heterozygous	c.1310G > C	p.Cys437Ser	Missense	Pathogenic	Novel mutation	‐
35	*DLL4*	Heterozygous	c.1397G > A	p.Cys466Tyr	Missense	Pathogenic	Novel mutation; *de novo*	‐
36	*DOCK6*	Compound heterozygous	c.484G > T	p.Glu162*	Nonsense	Pathogenic	d	Romani et al., 1998
			c.1362_1365delAACT	p.Thr455Serfs*24	Frameshift	Pathogenic	d, d	
37	*DOCK6*	Compound heterozygous	c.1362_1365delAACT	p.Thr455Serfs*24	Frameshift	Pathogenic	c, d	Sukalo et al., 2015 (Family 2)
			c.4491+1G > A	p.?	Splicing	Pathogenic	c	
38	*EOGT*	Homozygous	c.78_81delTCAC	p.His27Alafs*46	Frameshift	Pathogenic	Novel mutation	‐
39	*EOGT*	Homozygous	c.78_81delTCAC	p.His27Alafs*46	Frameshift	Pathogenic	Novel mutation	‐
22	*NOTCH1*	Heterozygous	c.1343G > A	p.Arg448Gln	Missense	Pathogenic	c; *de novo*	Southgate et al., 2015 (Family 5)
23	*NOTCH1*	Heterozygous	c.1343G > A	p.Arg448Gln	Missense	Pathogenic	c	Southgate et al., 2015 (Family 11); Girard et al., 2005 (Patient 2)
24	*NOTCH1*	Heterozygous	c.1345T > C	p.Cys449Arg	Missense	Pathogenic	c; *de novo*	Southgate et al., 2015 (Family 6)
25	*NOTCH1*	Heterozygous	c.1367G > A	p.Cys456Tyr	Missense	Pathogenic	c; *de novo*	Southgate et al., 2015 (Family 10); Girard et al., 2005 (Patient 1)
21	*NOTCH1*	Heterozygous	c.1393G > A	p.Ala465Thr	Missense	Likely pathogenic	Novel mutation	‐
26	*NOTCH1*	Heterozygous	c.1669+5G > A	p.?	Splicing e	Pathogenic	Novel mutation	‐
27	*NOTCH1*	Heterozygous	c.2380G > T	p.Glu794*	Nonsense	Pathogenic	Novel mutation	‐
15	*NOTCH1*	Heterozygous	c.2704C > T	p.Arg902Cys	Missense	Pathogenic	Novel mutation	*‐*
49	*NOTCH1*	Heterozygous	c.3281G > A	p.Cys1094Tyr	Missense	Pathogenic	Novel mutation	*‐*
198	*NOTCH1*	Heterozygous	c.4222G > T	p.Glu1408*	Nonsense	Pathogenic	Novel mutation; *de novo*	‐
183	*NOTCH1*	Heterozygous	c.4549G > A	p.Asp1517Asn	Missense	Likely pathogenic	Novel mutation; *de novo*	Verdyck et al., 2003 (Family 8)
28	*NOTCH1*	Heterozygous	c.4739dup	p.Met1580Ilefs*30	Frameshift	Pathogenic	c	Southgate et al., 2015 (Family 8)

a GenBank reference sequence and version number for *ARHGAP31*: NM_020754.3; *DLL4*: NM_019074.3; *DOCK6*: NM_020812.3; *EOGT*: NM_001278689.1; *NOTCH1*: NM_017617.4; *RBPJ*: NM_005349.3; numbering is from +1 as A of the ATG initiation codon.

b This column refers to medical case reports in which clinical features observed in specific families are described.

c Mutation was found in the same family as described in this publication.

d Mutation published previously in a different family.

e Exon skipping was verified at the RNA level.

We observed a causal mutation in 13 of the 36 families (36%) with likely autosomal recessive inheritance. These include homozygous mutations in *EOGT* (n = 3) and *DOCK6* (n = 7), and compound heterozygous mutations (*EOGT*, *n* = 1; *DOCK6*, *n* = 2). One additional case harbored a heterozygous VUS in *DOCK6*, but we did not detect a variant on the second allele (Supporting Information Table S3). The remaining 22 recessive families remain unresolved after analyzing all currently known AOS genes.

In the autosomal dominant cohort, 36% (20/55) of AOS cases were directly attributable to the established AOS genes with pathogenic mutations observed in *ARHGAP31* (*n* = 4), *DLL4* (*n* = 5), *NOTCH1* (*n* = 7), and *RBPJ* (*n* = 4) (Table 1). Furthermore, we observed four VUS in *NOTCH1* (Supporting Information Table S3). In one proband (Family 16), two *NOTCH1* missense variants were detected (p.Trp2034Arg and p.Ala2043Val). Both variants were confirmed to exist on the same allele, due to co‐occurrence in the same next‐generation sequencing read. These variants were not present in the healthy mother and sister. Due to the unavailability of paternal DNA, we were unable to determine whether either of these variants had occurred *de novo*.

In our cohort of 103 sporadic cases, the frequency of identified likely pathogenic mutations was 24%. We detected heterozygous mutations in *ARHGAP31* (*n* = 2), *DLL4* (*n* = 7), and *NOTCH1* (*n* = 12), in addition to compound heterozygous *DOCK6* mutations (*n* = 2) and homozygous *EOGT* mutations (*n* = 2) (Table 1). Additionally, we observed several VUS in *NOTCH1* (*n* = 6), *DLL4* (*n* = 3), and *DOCK6* (*n* = 1) (Supporting Information Table S3).

Taken together, mutations in the six established AOS genes underlie less than one third of the AOS/ACC/TTLD probands in our total cohort (Figure 1B). *NOTCH1* is the major contributor to the AOS phenotype, both in familial and sporadic disease, harboring 10% of the mutational load in our study. Mutations in *DLL4* and *DOCK6* each represent 6% of the cases while *ARHGAP31*, *EOGT*, and *RBPJ* mutations account for only small proportions, underlying 3%, 3%, and 2% of cases, respectively. Among familial cases we observed an elevated mutation detection rate of 36% overall (Figure 1B). *NOTCH1*, *DLL4*, *EOGT*, and *DOCK6* harbored deleterious variation across the major mutational categories, including insertion–deletion, nonsense, splicing, and missense variants (Figure 1C). By contrast, mutations observed in *ARHGAP31* comprised protein‐truncating mutations confined to the last exon, while all *RBPJ* mutations result in amino acid substitutions within a conserved DNA‐binding domain (Figure 1C).

An analysis of clinical features in our cohort determined that 96% of the mutation‐positive cases had scalp defects (with or without underlying skull defect), while TTLD was observed in 78% of mutation‐positive cases (Table 2). Assessment of potential genotype–phenotype correlations revealed wide variability in TTLD characteristics both within and between families. Brachydactyly and hypoplastic digits or nails were observed most frequently. ACC also demonstrated wide phenotypic variability, ranging from small patches of skin lacking hair to complete absence of skin with underlying skull defect. However, there did not appear to be any gene‐specific correlation with observed limb or scalp defects.

**Table 2:** Phenotype of mutation‐positive AOS patients

Fam ID	Gene	ACC (HP:0007385)	TTLD	Cardiac features (HPO id)	Vascular features (HPO id)	Other (HPO id)	Reference of family^a^
*Pedigrees suggestive of autosomal recessive inheritance*:							
4	*DOCK6*	+	+	?	?	?	Sukalo et al., 2015 (Family 8)
6	*DOCK6*	+	+	?	CMTC (HP:0000965)	IUGR (HP:0001511), brain abnormalities (HP:0000707), microcephaly (HP:0000252), ocular anomalies (HP:0000478), cognitive impairment (HP:0100543), epilepsy (HP:0001250), cerebral palsy (HP:0100021), abdominal skin defect	Sukalo et al., 2015 (Family 3)
8	*DOCK6*	+	+	?	?	Microcephaly (HP:0000252), ventricular dilatation/brain atrophy (HP:0002119; HP:0012444), corpus callosum hypoplasia/atrophy (HP:0007370), periventricular lesions (HP:0002518)	Sukalo et al., 2015 (Family 10)
9	*DOCK6*	+	+	−	?	Microcephaly (HP:0000252), ocular anomalies (HP:0000478), developmental delay (HP:0001263), epilepsy (HP:0001250), high palate (HP:0000218)	Sukalo et al., 2015 (Family 1)
10	*DOCK6*	+	+	−	?	IUGR (HP:0001511), microcephaly (HP:0000252), brain abnormalities (HP:0000707), ocular anomalies (HP:0000478), cognitive impairment (HP:0100543), epilepsy (HP:0001250), cryptorchidism (HP:0000028)	Sukalo et al., 2015 (Family 5); Prothero et al., 2007
11	*DOCK6*	+	+	PDA (HP:0001643)	?	Brain abnormalities (HP:0000707), microcephaly (HP:0000252), ocular anomalies (HP:0000478), knee dislocation (HP:0004976)	Sukalo et al., 2015 (Family 4)
13	*DOCK6*	+	+	?	?	Periventricular lesions (HP:0002518)	Sukalo et al., 2015 (Family 9)
5	*DOCK6*	+	+	VSD (HP:0001629)	?	IUGR (HP:0001511), microcephaly (HP:0000252), brain abnormalities (HP:0000707), ocular anomalies (HP:0000478), cognitive impairment (HP:0100543), epilepsy (HP:0001250), cerebral palsy (HP:0100021), abdominal skin defects, patella defects (HP:0003045)	Sukalo et al., 2015 (Family 6); Orstavik et al., 1995
7	*DOCK6*	+	+	TAPVD (HP:0005160)	?	IUGR (HP:0001511), microcephaly (HP:0000252), brain abnormalities (HP:0000707), ocular anomalies (HP:0000478), cognitive impairment (HP:0100543), epilepsy (HP:0001250), abdominal skin defect, hypothyroidism (HP:0000821)	Sukalo et al., 2015 (Family 7)
1	*EOGT*	+	+	?	CMTC (HP:0000965)	No other features; normal intelligence	−
2	*EOGT*	+	−	?	?	?	−
3	*EOGT*	+	+	−	CMTC (HP:0000965)	−	Temtamy et al., 2007 (Family 2)
59	*EOGT*	+	+	?	?	Cryptorchidism (HP:0000028) and small penis (HP:0000054), severe developmental delay (HP:0001263)	Verdyck et al., 2003 (Family 4)
*Pedigrees suggestive of autosomal dominant inheritance*:							
40	*ARHGAP31*	+	+	‐	?	−	Southgate et al., 2011 (Family AOS‐12); Bonafede & Beighton, 1979
41	*ARHGAP31*	?	?	?	?	?	−
42	*ARGHAP31*	‐	+	−	−	−	Isrie et al., 2014
43	*ARHGAP31*	+	+	−	?	−	Southgate et al., 2011 (Family AOS‐5); Verdyck et al., 2006
55	*DLL4*	+	−	−	?	?	Meester et al., 2015 (Family 6)
56	*DLL4*	+	+	?	CMTC (HP:0000965), portal hypertension (HP:0001409), esophageal varices (HP:0002040)	Epilepsy (HP:0001250), learning difficulties, mild periventricular leukomalacia (HP:0006970), splenomegaly (HP:0001744), congenital liver fibrosis (HP:0002612)	Meester et al., 2015 (Family 5)
57	*DLL4*	+	−	?	?	?	Meester et al., 2015 (Family 3)
58	*DLL4*	+	+	VSD (HP:0001629), tricuspid valve insufficiency (HP:0005180)	?	?	Meester et al., 2015 (Family 1)
181	*DLL4*	+	+	?	CMTC (HP:0000965)	?	−
14	*NOTCH1*	+	+	Aortic stenosis (HP:0001650)	?	?	−
46	*NOTCH1*	+	−	?	?	?	−
47	*NOTCH1*	+	+	Aortic and pulmonary valve abnormalities (HP:0001646; HP:0001641)	CMTC (HP:0000965)	−	Southgate et al., 2015 (Family 1), Verdyck et al., 2003 (Family 2)
48	*NOTCH1*	+	+	Pulmonic stenosis (HP:0001642)	?	IUGR (HP:0001511), hypoplastic mandible (HP:0000347), cerebral cortical dysplasia (brain abnormalities) (HP:0000707)	Savarirayan et al., 1999
50	*NOTCH1*	+	+	?	CMTC (HP:0000965)	−	Southgate et al., 2015 (Family 3)
52	*NOTCH1*	+	+	Aortic stenosis (HP:0001650), CoA (HP:0001680), Parachute mitral valve (HP:0011571), VSD (HP:0001629)	?	Long palpebral fissures (HP:0000637)	Southgate et al., 2015 (Family 4)
54	*NOTCH1*	+	−	CoA (HP:0001680), BAV (HP:0001647), parachute mitral valve (nonstenotic with mild regurgitation) (HP:0011571)	?	−	Southgate et al., 2015 (Family 2); Dallapiccola et al. 1992 (Patient 2)
44	*RBPJ*	+	+	TOF (HP:0001636)	CMTC (HP:0000965), pulmonary branch stenosis	Brain abnormalities (HP:0000707)	−
175	*RBPJ*	−	+	Severe and complex malformative cardiopathy (HP:0001627)	?	Digestive malrotation (HP:0002566)	−
45	*RBPJ*	+	+	?	?	Microcephaly (HP:0000252), hip dislocation (HP:0002827)	−
178	*RBPJ*	+	+	Secundum ASD (HP:0001631) and partial anomalous pulmonary venous drainage (HP:0010773)	−	−	−
*Sporadic probands*:							
17	*ARHGAP31*	?	?	?	?	?	−
18	*ARHGAP31*	+	+	?	?	−	−
30	*DLL4*	+	+	Truncus arteriosus (HP:0001660), VSD (HP:0001629)	?	Growth hormone deficiency (HP:0000824)	Meester et al., 2015 (Family 8)
31	*DLL4*	+	+	?	Died from bleeding left sinus transversus	?	−
32	*DLL4*	+	−	?	?	?	Meester et al., 2015 (Family 9)
182	*DLL4*	+	+	TOF (HP:0001636), absent pulmonary valve (HP:0005134)	Mild CMTC on thorax (HP:0000965), esophageal varices (HP:0002040), absent portal vein with portal hypertension (HP:0001409)	?	−
34	*DLL4*	+	−	?	?	?	Meester et al., 2015 (Family 4)
196	*DLL4*	+	+	TOF (HP:0001636)	?	Preterm birth (HP:0001622), cystic leukomalacia, bilateral hypoplasia of optic nerves (HP:0000609), secondary microcephaly (HP:0005484), died from multiorgan failure after heart surgery	−
35	*DLL4*	+	−	Long QT (HP:0001657)	?	Club feet (HP:0001762), brain abnormalities (leukomalacia), microcephaly (HP:0000252), cognitive impairment (HP:0100543)	−
36	*DOCK6*	+	+	−	?	IUGR (HP:0001511), brain abnormalities (HP:0000707), spasticity (HP:0001257), visual deficit (HP:0000505), intracranial calcifications (HP:0005671), global dilatation of ventricular system (HP:0002119)	Romani et al., 1998
37	*DOCK6*	+	+	?	CMTC (HP:0000965), single umbilical artery (HP:0001195)	Brain abnormalities (HP:0000707), microcephaly (HP:0000252), ocular anomalies (HP:0000478), cognitive impairment (HP:0100543), epilepsy (HP:0001250), cerebral palsy (HP:0100021), cryptorchidism (HP:0000028)	Sukalo et al., 2015 (Family 2)
38	*EOGT*	+	−	?	?	?	‐
39	*EOGT*	+	−	?	?	?	‐
22	*NOTCH1*	+	−	VSD (HP:0001629), pulmonary atresia (HP:0004935), right MBTS, Rastelli correction	Portal vein thrombosis, portal hypertension (HP:0001409)	Cognitive impairment (HP:0100543), T‐cell lymphopenia (HP:0001888), complex learning disability, autism (HP:0000717)	Southgate et al., 2015 (Family 5)
23	*NOTCH1*	+	+	?	Large hepatofugal coronary vein, tiny hepatoportal cavernoma, portal hypertension (HP:0001409)	Obliterative portal venopathy, hepatosplenomegaly (HP:0001433)	Southgate et al., 2015 (Family 11); Girard et al., 2005 (Patient 2)
24	*NOTCH1*	+	+	PTA (HP:0001660), VSD (HP:0001629)	?	−	Southgate et al., 2015 (Family 6)
25	*NOTCH1*	+	+	ASD (HP:0001631)	CMTC (HP:0000965), hepatopetal and hepatofugal collateral veins, portal hypertension (HP:0001409)	Obliterative portal venopathy, hepatosplenomegaly (HP:0001433)	Southgate et al., 2015 (Family 10); Girard et al., 2005 (Patient 1)
21	*NOTCH1*	+	+	?	CMTC (HP:0000965), missing portal vein	−	−
26	*NOTCH1*	+	+	VSD (HP:0001629)	CMTC (HP:0000965)	−	−
27	*NOTCH1*	+	+	?	Sinus sagittalis thrombosis	?	−
15	*NOTCH1*	?	?	?	?	?	−
49	*NOTCH1*	+	+	−	CMTC (HP:0000965)	−	−
198	*NOTCH1*	+	−	−	CMTC (HP:0000965)	Normal brain	−
183	*NOTCH1*	+	+	−	?	Full body CT normal, brain scan normal. Mildly delayed motor skills (HP:0002194)—walked at 15 months, slow speech—otherwise normal development	Verdyck et al., 2003 (Family 8)
28	*NOTCH1*	+	+	−	CMTC (HP:0000965)	Epilepsy (HP:0001250), dyslexia (HP:0010522)	Southgate et al., 2015 (Family 8)

ACC, aplasia cutis congenita; AS, aortic stenosis; ASD, atrial septal defect; AV, aortic valve; CHD, congenital heart defect; CMTC, cutis marmorata telangiectatica congenita; CoA, coarctation of the aorta; CT, Computed Tomography; IUGR, intrauterine growth restriction; MBTS, modified Blalock‐Taussig shunt; MV, mitral valve; PH, portal hypertension; PDA, patent ductus arteriosus; PTA, persistent truncus arteriosus; PV, pulmonary valve; TAPVD, total anomalous pulmonary venous drainage; TOF, Tetralogy of Fallot; TTLD, terminal transverse limb defects; TVI, tricuspid valve insufficiency; VSD, ventricular septal defect; ?, unknown; −, absent; +, present

a This column refers to medical case reports in which clinical features observed in specific families are described.

We observed a wide variety of associated cardiac features, including ASD, VSD, patent ductus arteriosus, aortic stenosis, truncus arteriosus, TOF, and valve abnormalities. Of note, cardiac features were more frequently observed in patients with a mutation in *DLL4, NOTCH1*, or *RBPJ* (≥49% vs. ≥13%, Table 2). However, in the absence of detailed cardiac examinations for all variant carriers, it was not possible to determine firm genotype–phenotype correlations based on these data. CMTC was reported in 29% of mutation‐positive patients, while other observed vascular features included defects of pulmonary or portal vasculature, abnormal branching of the carotid artery, and sinus sagittalis thrombosis (Table 2). In *DOCK6*‐positive cases, we observed a positive correlation with the presence of brain abnormalities and/or intellectual disability, as previously described (≥91% vs. ≥19%, Table 2) (Sukalo et al., 2015).

## 4 DISCUSSION

Here, we have examined the genetic contribution to AOS and isolated ACC/TTLD in our extensive cohort of families ascertained through the European AOS Consortium. With the discovery of 63 mutations in the six previously established genes, including 56 distinct and 22 novel mutations, our study provides independent confirmation of a substantial role for *ARHGAP31*, *DLL4*, *DOCK6*, *EOGT*, *NOTCH1*, and *RBPJ* in AOS pathogenesis. This combined mutation screening strategy represents the largest cohort of AOS patients reported to date and, while some of the cases detailed here have been previously published in cohorts used for novel gene identification (Table 1), this comprehensive review and mutation update provides unique insight into the distribution and frequency of mutations across the wider spectrum of AOS‐related disorders. The majority of identified mutations (*n* = 41; 71%) affect genes within the Notch pathway and are therefore predicted to lead to dysregulated Notch signaling, likely through haploinsufficiency or loss‐of‐function (LOF) of NOTCH1, DLL4, RBPJ, or EOGT. A smaller proportion (*n* = 17; 29%) affects the Rho GTPase regulators ARHGAP31 and DOCK6, which specifically influence the activity of RAC1 and CDC42.

### 4.1 Autosomal dominant AOS

Consistent with previous reports, our data confirm that *NOTCH1* is the major contributor to the genetic basis of autosomal dominant AOS/ACC/TTLD (Stittrich et al., 2014). In addition to 9 previously reported variants (Supporting Information Table S4) (Southgate et al., 2015), we identified 10 novel mutations and nine VUS in the *NOTCH1* gene (Figure 1D; Supporting Information Table S3). Protein‐truncating variants are distributed across the length of the receptor and are predicted to lead to nonsense‐mediated decay (NMD) of the mutant mRNA transcript. In contrast, and as discussed previously (Southgate et al., 2015), we observed a clustering of missense *NOTCH1* mutations around EGF‐like domains 11–13, critical for ligand binding to the receptor (Hambleton et al., 2004; Luca et al., 2015). Here, we describe one novel missense mutation (p.Ala465Thr) within EGF12, which has been reported in ClinVar as likely pathogenic for an unspecified condition. We also identified a novel splice‐site mutation (c.1669+5G > A), confirmed by cDNA sequencing to lead to in‐frame skipping of exon 10, encoding residues within EGF13–14 (Supporting Information Figure S2 and Supporting Information Table S2). This variant has a minor allele frequency (MAF) of 4 × 10^−6^ in the gnomAD control database (https://gnomad.broadinstitute.org/; V.r2.0.2). The identification of these mutations in our AOS cohort provides further confirmation of the importance of this ligand‐binding region for normal human development. We also describe five cysteine‐replacing or ‐creating mutations within other EGF‐like repeat domains, of which two (p.Arg902Cys and p.Cys1094Tyr) are novel. Cysteine residues within this region form essential disulfide bonds (Dietz et al., 1992; Schrijver et al., 1999), suggesting that these mutations will most likely disrupt the tertiary structure of these domains. A number of additional missense variants were classified as VUS due to the lack of familial segregation data. Of note, the proband in Family 16 harbored two missense variants within the highly conserved ANK4 protein domain, essential for RBPJ binding (Aster et al., 2000). Taken with reports of *RBPJ* LOF in AOS, these findings provide a strong indication that one of these two variants is likely pathogenic.

We observed four novel mutations in *DLL4*, the majority of which are missense, including two cysteine substitutions (p.Cys437Ser and p.Cys466Tyr) and a substitution in EGF‐like domain 3 (p.Thr317Pro). We additionally identified a novel nonsense mutation (p.Gln609*) and a c.572G > A (p.Arg191His) missense mutation, recently reported in a Japanese family (Nagasaka et al., 2017). The Arg191 residue is a highly conserved residue in the DSL domain of DLL4, which stabilizes receptor–ligand binding with the NOTCH1 EGF12 domain (Luca et al., 2015). The VUS identified in *DLL4* include one missense variant (p.Pro267Thr), which has been reclassified here according to ACMG guidelines (Richards et al., 2015), an in‐frame deletion (p.Phe89delCTT), and a potential splice‐site variant (c.1240+5G > C) (Supporting Information Table S2). The observed spectrum of *DLL4* variation, including those identified previously (Supporting Information Table S4), suggests that LOF is the likely molecular mechanism in *DLL4*‐positive AOS; however, this remains to be experimentally verified.

In accordance with the previous report of *RBPJ* substitutions within critical DNA‐binding domains (Supporting Information Table S4) (Hassed et al., 2012), we identified four DNA‐binding domain missense mutations in *RBPJ*, one of which (p.Lys169Glu) is recurrent, providing further evidence for LOF of this transcription factor in AOS pathogenesis. *RBPJ* is highly intolerant to variation, with no LOF and few missense variants observed in ExAC (https://exac.broadinstitute.org; V.0.3.1). We detected two novel substitutions within critical DNA‐binding domains. The p.Arg65Gly mutation in Family 44 affects an amino acid residue that has previously been demonstrated to bind directly to DNA (Hassed et al., 2012), while the p.Phe66Val substitution (Family 175) is considered likely pathogenic due to its position, evolutionary conservation and familial segregation. In this family, a *NOTCH1* variant (p.Arg1758Gly) was observed to cosegregate with disease, but was classified as a VUS due to poor conservation and its presence in control databases (MAF in gnomAD = 0.00014). We additionally identified a novel p.Ser332Arg missense substitution in Family 178 (Supporting Information Table S1). Due to ambiguity around the exact location of RBPJ domain predictions, it is unclear whether this substitution is located within the beta‐trefoil DNA‐binding domain; however, all *RBPJ* variants are consistent with a LOF disease mechanism due to likely abrogation of DNA binding.

Pathogenic mutations in *ARHGAP31* reported to date (Supporting Information Table S4) are all located within the terminal exon 12, leading to premature termination of the translated protein (Isrie, Wuyts, Van Esch, & Devriendt, 2014; Southgate et al., 2011). Mutation screening of *ARHGAP31* in our cohort identified four heterozygous protein‐truncating mutations within exon 12. In addition to one novel nonsense mutation (p.Gln728*), we detected a recurrent p.Gln683* mutation in three unrelated cases, highlighting terminal exon termination mutations as a consistent feature of this gene in AOS. Microsatellite genotyping to assess potential shared haplotypes at this locus was inconclusive (data not shown). Previous analysis of the p.Gln683* mutation has demonstrated the mutant transcript escapes NMD, consistent with truncating mutations downstream of the final splice junction (Bonafede & Beighton, 1979), leading to the production of a constitutively active protein and disruption of the actin cytoskeleton due to active CDC42 depletion (Southgate et al., 2011). We therefore hypothesize that other C‐terminal protein‐truncating mutations of ARHGAP31 will lead to a gain of protein function through a similar mechanism of NMD escape.

### 4.2 Autosomal recessive AOS

In our autosomal recessive cohort, mutation analysis of the *EOGT* gene revealed five distinct mutations across six families. All variants have a very low frequency in gnomAD (maximum MAF = 0.00002) and, with the exception of one family, were present in the homozygous state. In two Dutch families (Family 38 and 39), we observed an identical novel homozygous frameshift mutation (p.His27Alafs*46). Although present in the heterozygous state in gnomAD (MAF = 0.00005), no homozygous genotypes were observed in European control populations. In a third family from the United Kingdom (Family 59), this mutation was present in compound heterozygosity with a second causative variant. While we were unable to formally evaluate relatedness between these families, it is notable that a recurrent *EOGT* mutation exists within distinct European populations.

We identified a total of 13 distinct *DOCK6* mutations and two VUS in *DOCK6*, all of which have been previously reported by our consortium (Supporting Information Table S4) (Sukalo et al., 2015). As expected, all homozygous mutations were present in families with close parental relatedness. In our cohort of compound heterozygous mutations, four variants (c.484G > T, c.788T > A, c.1902_1905delGTTC, and c.4106+5G > T) are unique to this consortium, while three variants have a low MAF in gnomAD (c.5939+2T > C; 0.00021, c.1362_1365del; 0.00004, and c.4491+1G > A; 0.00003). In two families, we only observed a single heterozygous low frequency variant. Identification of a second mutation, for example, within the noncoding region at a cryptic intronic splice site, enhancer/repressor region, or promoter region, could provide a molecular diagnosis in a limited set of patients.

Of interest, a few consanguineous families remain genetically unresolved after analyzing the coding region of the six established AOS genes. Due to the methodology used, we were unable to detect larger structural variation in these genes, which may explain a proportion of the missing heritability in AOS. Of note, linkage analysis in one consanguineous family demonstrated autozygosity across the *EOGT* locus but no coding mutation was identified in this gene. In several other families, genome‐wide autozygosity mapping analysis has indicated the likely existence of other disease loci that require further investigation. These data strongly suggest that there are still more genes to be discovered in AOS/ACC/TTLD.

### 4.3 Genetic architecture

A comparison of the proportion of AOS cases attributable to each particular gene observed in our current study (Figure 1B) against those previously published (Lehman et al., 2016) reveals a number of key differences. Specifically, we observed a lower frequency of mutations for all genes in our cohort. Our results indicate an overall diagnostic yield of 30%, compared to the previous report that 50%–60% of AOS cases are explained by mutations in the six established genes. This may be due to previous estimates being largely based on single reports of novel gene identification, which typically utilize highly stratified discovery cohorts that are both clinically homogeneous and mutation negative for previously characterized genes. The latter would therefore lead to an overestimation of the number of identified mutations. Of note, our cohort contains a significant number of cases and families with isolated ACC or TTLD. Given recent observations of a wide phenotypic spectrum in AOS mutation carriers, the updated diagnostic criteria proposed in 2016 (Lehman et al., 2016), which account for the presence of a pathogenic mutation in an established gene, may be more valid, leading to reclassification of nine ACC cases and one TTLD case as AOS in our cohort (data not shown). While the removal of isolated ACC/TTLD cases from our cohort does not significantly alter the diagnostic yield, it is notable that the mutation‐negative sporadic cohort contains predominantly isolated ACC cases (*n* = 27). Additionally, although we have used robust diagnostic methodologies, it is possible that our cohort may contain some cases with other genetic or nongenetic conditions.

An alternative explanation for the difference in mutation frequency between the two reports is the use of more stringent criteria for classification of identified variants in our study. We classify several variants as VUS, due to strict adherence to the ACMG guidelines. However, functional evaluation or familial segregation analyses may alter these classifications. For example, the NOTCH1 p.Asp1989Asn variant has previously been classified as pathogenic (Stittrich et al., 2014), but has since been reclassified as benign in ClinVar. Similarly, we have reclassified the NOTCH1 p.Pro407Arg and DLL4 p.Pro267Thr variants as VUS in this study (Meester et al., 2015; Southgate et al., 2015). Of note, a reclassification of all the VUS in our study as causal, would increase the diagnostic yield to 36% (Figure 1B), which is still lower than previous estimates (Lehman et al., 2016). By contrast, the use of additional gene‐specific criteria for classification, as described above, may have led to an overrepresentation of pathogenic variants.

Finally, our study includes a substantial proportion of sporadic cases, which have been relatively poorly studied in previous reports favoring the use of familial cohorts for novel gene detection. A molecular diagnosis was achieved for 36% of the familial cases in our cohort, or 40% if classifying all VUS as causal (Figure 1B). Conversely, in our sporadic cases the mutation detection rate was only 24%, highlighting an increased likelihood of genetic risk factors in familial disease. Our sporadic cohort also contains nine, predominantly missense VUS. These variants would require additional supporting evidence to be reclassified as pathogenic. However, this is complicated by the absence of familial segregation data and documented reduced penetrance in this condition.

An assessment of potential genotype–phenotype correlations in our cohort revealed a few important observations. Cardiac features were more frequently observed in patients with a mutation in *NOTCH1, DLL4*, or *RPBJ*. While cardiac examination is recommended for all AOS patients, these findings indicate a specific requirement for patients with *NOTCH1, DLL4*, and *RBPJ* mutations. We also noted a positive correlation between patients with recessive mutations in *DOCK6* and the presence of neurological abnormalities, intrauterine growth restriction, or ocular anomalies. Finally, we observed wide phenotypic variability and incomplete penetrance. The latter was most common in *NOTCH1*‐related AOS and to a lesser extent in *ARHGAP31*‐ and *DLL4*‐related disease. It is likely that the level of penetrance is currently overestimated, due to segregation analysis typically being restricted to parents. Furthermore, incomplete penetrance potentially accounts for an excess of sporadic cases, a known phenomenon in AOS.

### 4.4 Future perspectives

Despite the identification of six genes underlying AOS to date, the majority of cases (64%–70%) in our cohort remain unresolved. Several reasons that may explain this missing heritability should now be examined further. First, targeted next‐generation sequencing is dependent on efficient hybridization, which did not provide full coverage of all target genes. Specifically, *NOTCH1* exon 1 was poorly covered using HaloPlex Target enrichment and TruSeq Custom Amplicon enrichment. While these gaps were not sequenced manually, no AOS mutations have been reported in this region of the gene to date. In addition, several target regions of *DOCK6* (exons 1, 2, 15, 16, 23) and *RBPJ* (exon 2) demonstrated reduced coverage in a subset of patients enriched with the TruSeq Custom Amplicon Panel. Second, further genetic heterogeneity of AOS is highly likely. Additional genes encoding proteins involved in NOTCH signaling or CDC42/RAC1 regulation and cytoskeleton dynamics are plausible candidates. Third, it is possible that the spectrum of AOS/ACC/TTLD disease is not uniformly monogenic. Considering the high proportion of sporadic cases for a condition that does not significantly reduce reproductive fitness, as well as the likelihood that this spectrum of disorders is a consequence of fetal vascular disruption, it is tempting to speculate that nongenetic causes or complex inheritance may be involved in the etiology of this phenotype. Fourth, the majority of samples in our cohort have not undergone copy‐number variant analysis or screening of noncoding regions. Partial or complete deletion or duplication of one of the six established genes may account for approximately 20% of cases (Machado et al., 2015), but were not detected in this study due to the methodologies used. In conclusion, these data support the likely existence of additional, as yet unidentified, susceptibility genes for AOS and related disorders. Our extensive patient cohort provides opportunities for the identification of additional causal genes and functional interpretation of identified defects, with the potential to explore future therapeutic avenues in this condition.

## Supporting information

Supporting Information
