# Supplementary material for: Elucidating the genetic architecture of Adams–Oliver syndrome in a large European cohort
Source: Hum Mutat. 2018 Jul 4;39(9):1246–61. doi: 10.1002/humu.23567 (PMC6175364; doi:10.1002/humu.23567)
Supplement: Supplementary file 1 — Supporting Information [file HUMU-39-1246-s001.pdf]

Inventory of Supporting Information

List of clinicians who recruited patients to the European AOS Consortium
Supplementary Figure S1
Supplementary Figure S2
Supplementary Table S1
Supplementary Table S2
Supplementary Table S3
Supplementary Table S4
Supplementary references

List of clinicians who recruited patients to the European AOS Consortium

Jon Aase, Mona Aglan, Beate Albrecht, Lihadh Al-Gazali, Julia Barone, Sander Beekmans, Emilia Bijlsma, Bettina Blaumeiser, Mary-Louise Bonduelle, Francesco Brancati, Berten Ceulemans, Wiltrud Coerdt, Winnie Courtens, Carol Crowe, Bruno Dallapiccola, Marjan de Rademaeker, Maryse De Smedt, Bert de Vries, Nicolette den Hollander, Ellen Denayer, Charu Deshpande, Julie Desir, Koen Devriendt, Maria Cristina Digilio, Diana Dijkman, Joanne Dixon, Peter Don Griot, Laurence Fayol, Helen Firth, Chin-To Fong, Lilianne Gomez-Lopez, Vanesa López González, Luitgard Graul-Neumann, Ute Hehr, Blanca Hernández Charro, Simon Holden, Denise Horn, Judith Horvath, Irina Hüning, Jane Hurst, Emmanuel Jacquemin, Sandra Janssens, An Jespers, Ariana Kariminejad, Hülya Kayserili, Esra Kiliç, Esther Kinning, Merel Klaassens, Margarete Koch, Yves Lacassie, Wayne Lam, Anne Lampe, Pablo Lapunzina, Chumei Li, Sally Ann Linch, Edward Lose, Pilar Madero Barrajón, Mauro Maniscalco, Ertan Mayatepek, Leslie McGregor, Vardiella Meiner, Sandra Mercier, Klaus Mohnike, Michelle Moore, Dietmar Müller, Stefan Mundlos, Isabel Navarro, John Nelson, Karen Helene Ørstavik, Lilian Bomme Ousager, Millan Patel, Genevieve Pierquin, Lucile Pinson, Sabine Preis, Katrina Prescott, Trine Prescott, Natalija Pronina, Lluís Puig, Alessandra Renieri, Deborah Ruddy, Claudia Ruivenkamp, Cristina Rusu, Leonardo Salviati, Shaida Schirwani, Eva Seemanova, Debbie Shears, Vanja Slootmaekers, Wendy Smith, Guillaume Smits, Katie Snape, Gabriela Soares, Bernhard Steiner, Helen Stewart, Marijn Stribos, Yves Sznajer, Mustafa Tekin, Samia Temtamy, Sanne Traasdahl Møller, Beyhan Tüysüz, Michiel Vaandrager, Jenneke Van den Ende, Nathalie van der AA, Fleur van Dijk, Nicole van Regemorter, Anthony Vandersteen, Joke Verheij, Kristin Vinorum, Emma Wakeling, Franziska Waldmann, Astrid Weber, Marjolaine Willems, Patrick Willems, and Andrea Zonta.

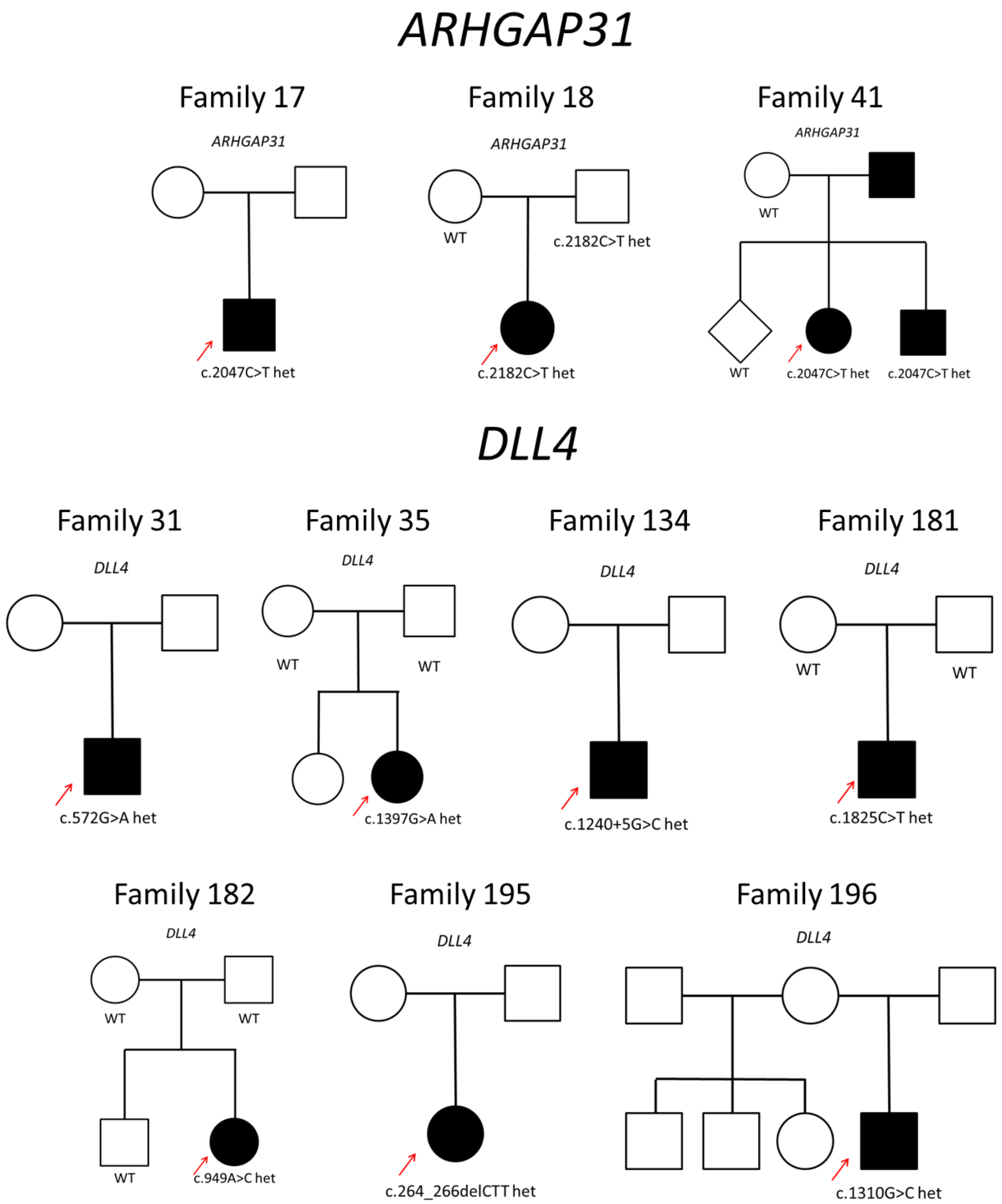

Figure S1. **Pedigrees of unpublished families with a novel mutation or a variant of uncertain significance.**

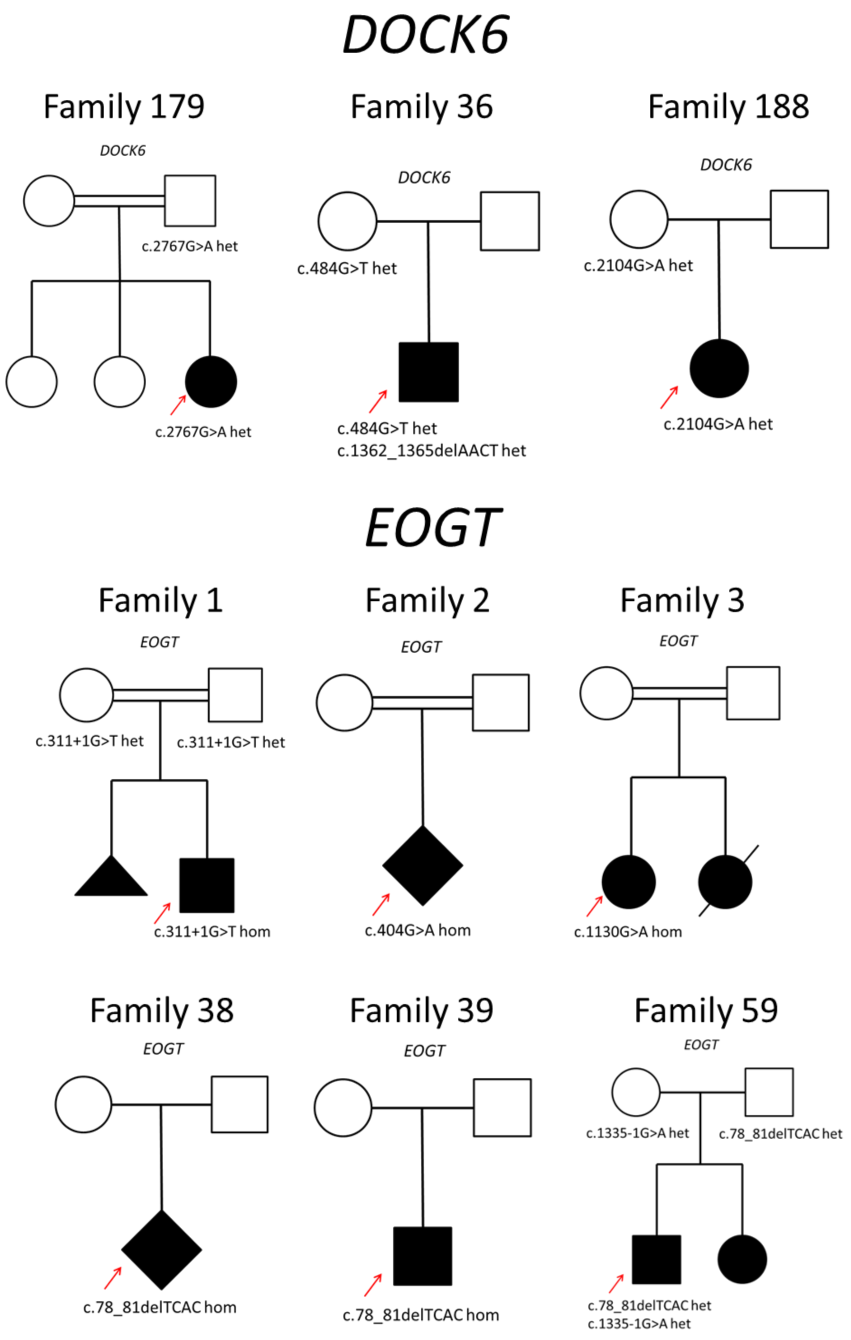

Figure S1. **Pedigrees of unpublished families with a novel mutation or a variant of uncertain significance** (continued)**.**

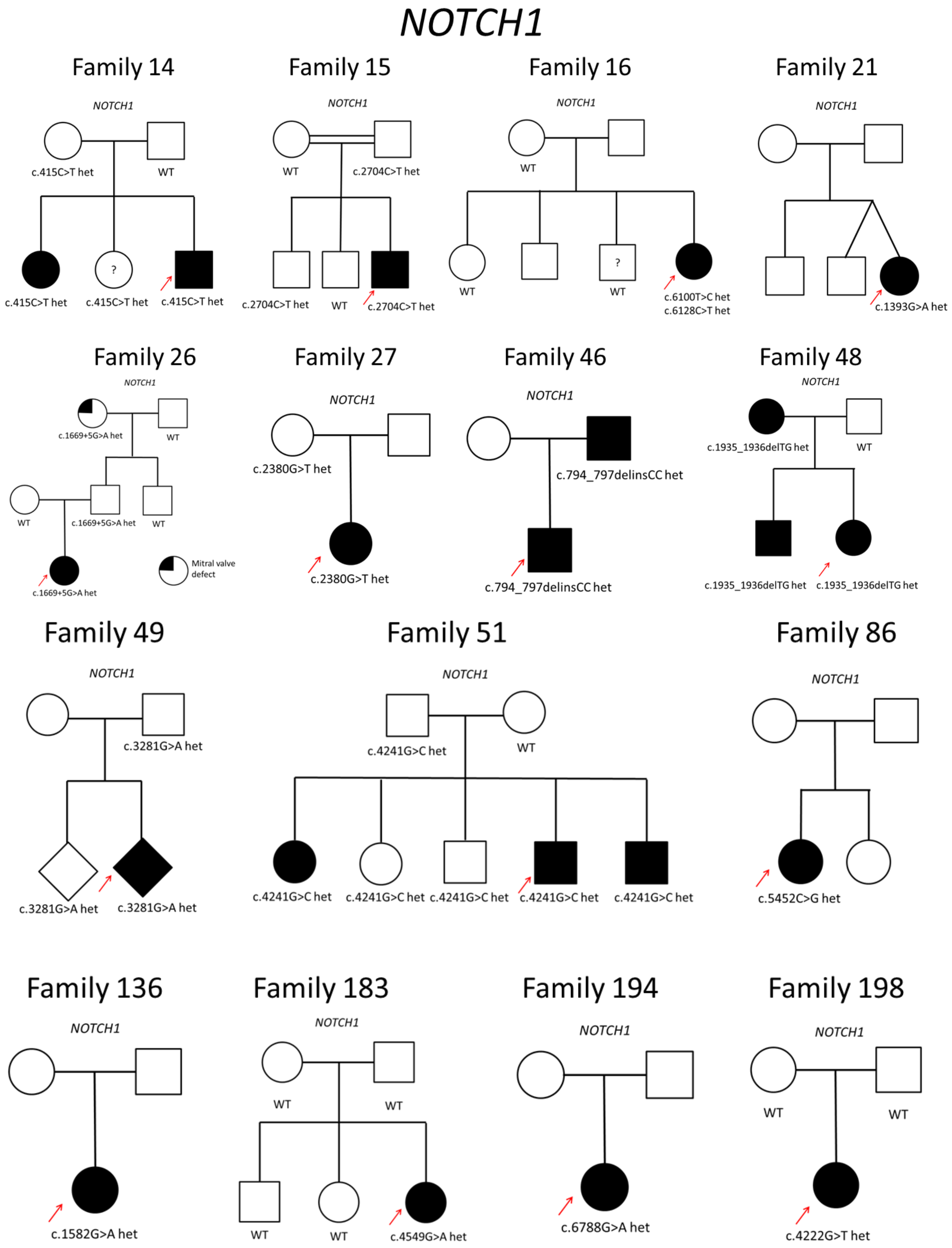

Figure S1. **Pedigrees of unpublished families with a novel mutation or a variant of uncertain significance** (continued)**.**

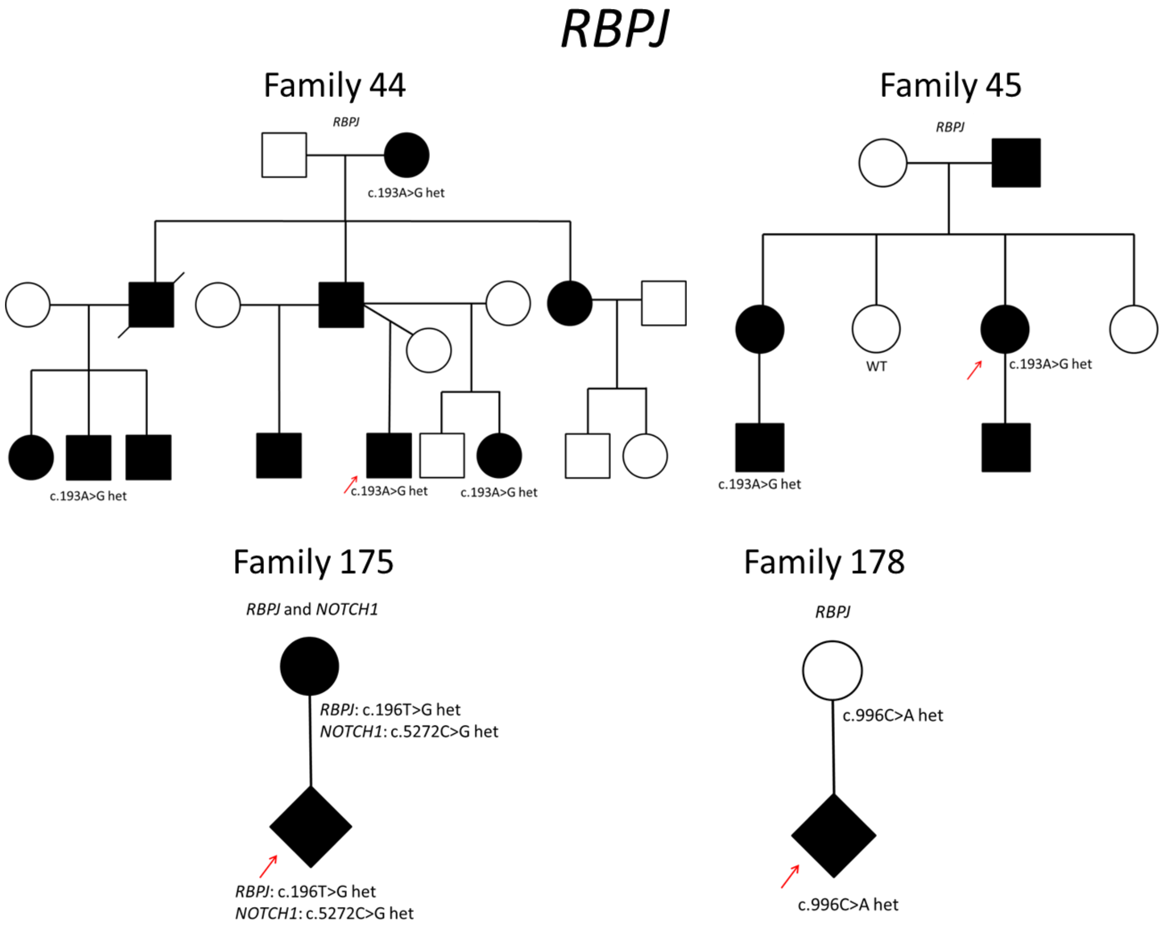

Figure S1. **Pedigrees of unpublished families with a novel mutation or a variant of uncertain significance** (continued)**.**

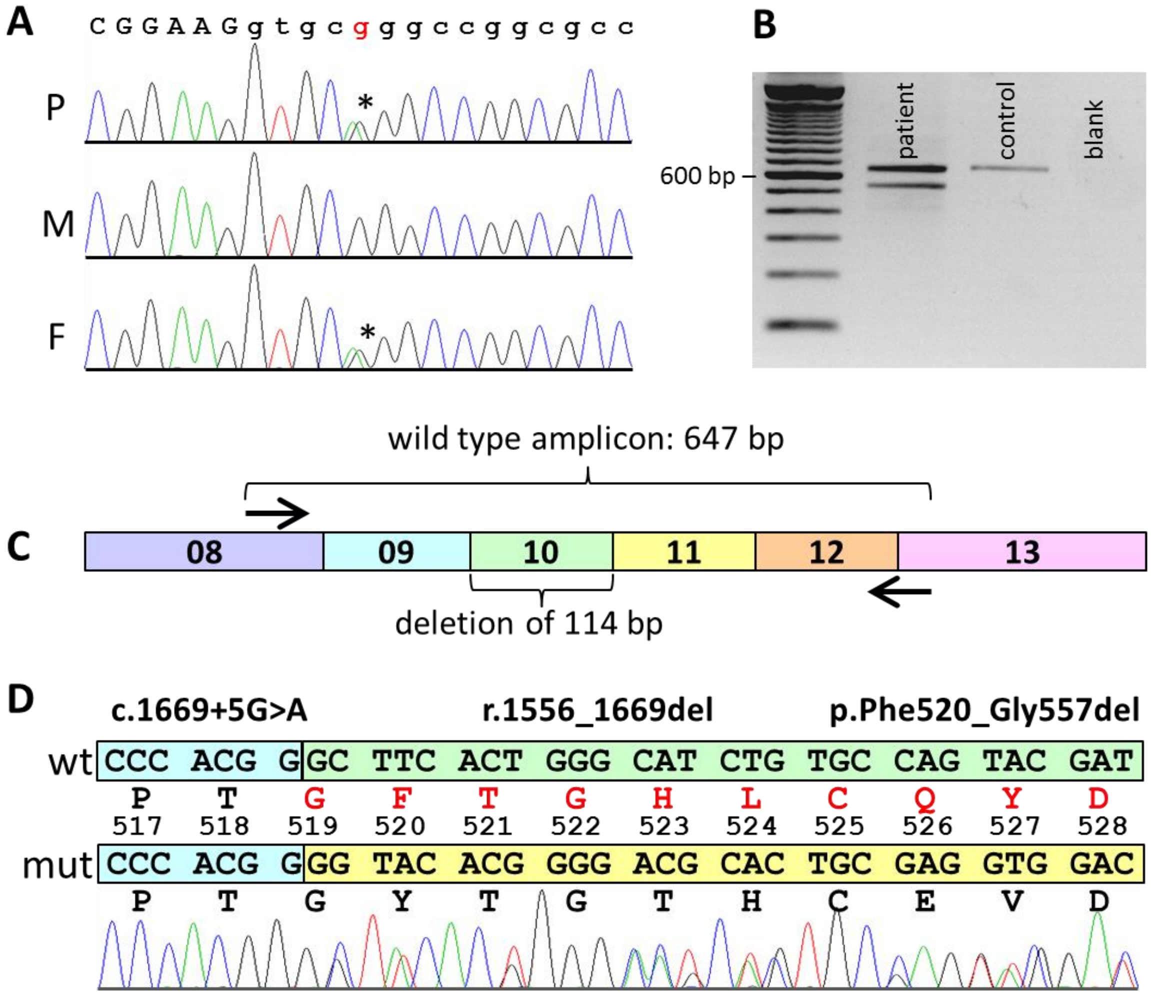

Figure S2. **Analysis of the splice site mutation c.1669+5G>A in *NOTCH1*.**

(**A**) Electropherograms showing the splice site mutation (*) on the genomic DNA level. Capital letters indicate exonic regions, intronic regions are written in lower case letters. P, patient; M, mother; F, father. (**B**) Agarose gel electrophoresis of RT-PCR products obtained from leukocyte RNA. The two bands in the patient represent a wild type allele (same as in the control) and a shorter fragment where the complete exon 10 (114 bp) is missing. (**C**) Position of RT-PCR primers and relative exon sizes. (**D**) Wild type cDNA sequence (wt) and amino acid residues are compared to the mutated cDNA sequences (mut) and encoded amino acid are given in the one-letter code. Wild type shows correct junction of exon 9 (blue box) and 10 (green box). cDNA from the patient contains the normal exon 9 – exon 10 junction as well as a junction of exon 9 and exon 11 (yellow box), thus confirming skipping of exon 10 due to the mutation c.1669+5G>A.

Table S1. ***In silico* predictions for missense variants.**

Gene	Nucleotide change [a]	Amino acid change	CADD	MutationTaster	SIFT	PolyPhen-2 hvar	gnomAD MAF	Pathogenicity class
DLL4	c.361G>C	p.Ala121Pro	27.60	D (1.000)	D	D (0.997)	-	Likely pathogenic
DLL4	c.556C>T	p.Arg186Cys	33.00	D (1.000)	D	D (0.963)	-	Likely pathogenic
DLL4	c.572G>A	p.Arg191His	35.00	D (1.000)	D	D (0.997)	-	Likely pathogenic
DLL4	c.583T>C	p.Phe195Leu	26.00	D (1.000)	D	D (0.985)	-	Likely pathogenic
DLL4	c.799C>A	p.Pro267Thr	29.40	D (1.000)	D	P (0.750)	-	VUS
DLL4	c.949A>C	p.Thr317Pro	25.40	D (1.000)	D	D (0.974)	-	Likely pathogenic
DLL4	c.1168T>C	p.Cys390Arg	27.00	D (1.000)	D	D (1.000)	-	Pathogenic
DLL4	c.1169G>A	p.Cys390Tyr	28.50	D (1.000)	D	D (1.000)	-	Pathogenic
DLL4	c.1310G>C	p.Cys437Ser	23.80	D (1.000)	D	D (0.999)	-	Pathogenic
DLL4	c.1365C>G	p.Cys455Trp	26.60	D (1.000)	D	D (0.975)	-	Pathogenic
DLL4	c.1397G>A	p.Cys466Tyr	26.50	D (1.000)	D	D (1.000)	-	Pathogenic
DOCK6	c.788T>A	p.Val263Asp	30.00	D (1.000)	D	D (0.998)	-	Likely pathogenic
DOCK6	c.2104G>A	p.Gly702Ser	23.70	D (0.777)	D	B (0.211)	0.002613	VUS
DOCK6	c.2767G>A	p.Val923Ile	15.21	D (1.000)	T	B (0.035)	0.000213	VUS
DOCK6	c.3047T>C	p.Leu1016Pro	27.70	D (1.000)	D	D (0.986)	-	Likely pathogenic
DOCK6	c.3154G>A	p.Glu1052Lys	34.00	D (1.000)	D	D (1.000)	-	Likely pathogenic
DOCK6	c.4786C>T	p.Arg1596Trp	29.80	D (1.000)	D	D (0.999)	-	Likely pathogenic
EOGT	c.404G>A	p.Cys135Tyr	32.00	D (1.000)	D	D (0.999)	0.000004	Pathogenic
EOGT	c.878G>A	p.Arg377Gln	27,60	D (1.000)	T	D (1.000)	0.000001	Likely pathogenic
NOTCH1	c.1220C>G	p.Pro407Arg	23.70	D (1.000)	T	D (0.929)	-	VUS
NOTCH1	c.1343G>A	p.Arg448Gln	34.00	D (1.000)	D	D (0.917)	-	Pathogenic
NOTCH1	c.1345T>C	p.Cys449Arg	27.40	D (1.000)	D	D (1.000)	-	Pathogenic
NOTCH1	c.1367G>A	p.Cys456Tyr	27.90	D (1.000)	D	D (1.000)	-	Pathogenic
NOTCH1	c.1393G>A	p.Ala465Thr	34.00	D (1.000)	D	P (0.907)	-	Likely pathogenic
NOTCH1	c.1582G>A	p.Asp528Asn	24.00	D (1.000)	T	B (0.322)	0.000082	VUS
NOTCH1	c.2704C>T	p.Arg902Cys	27.70	D (1.000)	T	D (0.932)	-	Pathogenic
NOTCH1	c.3281G>A	p.Cys1094Tyr	25.60	D (1.000)	D	D (0.998)	-	Pathogenic
NOTCH1	c.4120T>C	p.Cys1374Arg	25.30	D (1.000)	D	D (0.998)	-	Pathogenic
NOTCH1	c.4241G>C	p.Cys1414Ser	25.60	D (1.000)	D	D (0.997)	-	Pathogenic
NOTCH1	c.4549G>A	p.Asp1517Asn	31.00	D (1.000)	D	D (1.000)	-	Likely pathogenic
NOTCH1	c.5218G>T	p.Ala1740Ser	22.80	N (0.980)	D	B (0.093)	-	VUS
NOTCH1	c.5272C>G	p.Arg1758Gly	25.30	D (1.000)	T	P (0.741)	0.000004	VUS
NOTCH1	c.5452C>G	p.Leu1818Val	11.47	D (0.949)	T	B (0.276)	-	VUS
NOTCH1	c.6100T>C	p.Trp2034Arg	26.10	D (1.000)	D	D (1.000)	-	VUS
NOTCH1	c.6128C>T	p.Ala2043Val	26.50	D (1.000)	D	D (0.999)	-	VUS
NOTCH1	c.6788G>A	p.Arg2263Gln	17.30	D (1.000)	T	B (0.187)	0.000352	VUS
RBPJ	c.193A>G	p.Arg65Gly	25.80	D (1.000)	D	D (0.970)	-	Likely pathogenic
RBPJ	c.196T>G	p.Phe66Val	26.10	D (1.000)	D	D (0.970)	-	Likely pathogenic
RBPJ	c.505A>G	p.Lys169Glu	28.90	D (1.000)	D	D (0.969)	-	Pathogenic
RBPJ	c.996C>A	p.Ser332Arg	31.00	D (1.000)	D	D (1.000)	-	Likely pathogenic

Population frequency data were obtained from the gnomAD database (http://gnomad.broadinstitute.org/). Annovar (version dbnsfp30a) annotation was used for functional prediction scores, including MutationTaster, SIFT, PolyPhen2 hvar, and CADD. Default parameters were used for each *in silico* prediction method. Ranges and cut-offs for output scores were as follows. MutationTaster: range 0-1 (disease causing (D): >0.5); SIFT: range 0-1 (damaging (D): ≤0.05; tolerated (T): >0.05); PolyPhen-2 hvar: range: 0-1 (benign (B): 0-0.452; possibly damaging (P): 0.453-0.956; probably damaging (D): 0.957-1).

[a] GenBank reference sequence and version number for *ARHGAP31*: NM_020754.3; *DLL4*: NM_019074.3; *DOCK6*: NM_020812.3; *EOGT*: NM_001278689.1; *NOTCH1*: NM_017617.4; *RBPJ*: NM_005349.3; numbering is from +1 as A of the ATG initiation codon.

MAF, Minor Allele Frequency; VUS, variant of uncertain significance.

Table S2. ***In silico* splicing prediction.**

Gene	Nucleotide change [a]	SpliceSiteFinder-like	MaxEntScan	NNSPLICE	GeneSplicer	Human Splicing Finder
DLL4	c.1240+5G>C	-100%	-62%	-100%	-48%	-14%
DOCK6	c.4491+1G>A	-100%	-100%	-100%	-100%	-100%
DOCK6	c.5939+2T>C	=	-100%	-100%	-100%	-100%
DOCK6	c.4106+5G>T	-100%	-100%	-100%	-86%	-15%
EOGT	c.311+1G>T	-100%	-100%	-100%	=	-28%
EOGT	c.1335-1G>A	-7%	-76%	-100%	-100%	-9%
NOTCH1	c.1669+5G>A	-100%	-51%	-100%	-9%	-14%

Alamut Visual version 2.8.1. was used for splice site predictions of SpliceSiteFinder-like, MaxEntScan, NNSPLICE, GeneSplicer, and Human Splicing Finder. =, no predicted change of splice-site.

[a] GenBank reference sequence and version number for *DLL4*: NM_019074.3; *DOCK6*: NM_020812.3; *EOGT*: NM_001278689.1; *NOTCH1*: NM_017617.4; numbering is from +1 as A of the ATG initiation codon.

Table S3. **VUS in established AOS genes.**

Fam ID	Gene	Nucleotide change [a]	Amino acid change	Type of mutation	gnomAD (MAF) [b]	Mutation previously reported	Reference of family [c]	Comments
Pedigrees suggestive of autosomal recessive inheritance:								
179	*DOCK6*	c.2767G>A	p.Val923Ile	Missense	0.000213	[1]	-	Parental consanguinity
Pedigrees suggestive of autosomal dominant inheritance:								
51	*NOTCH1*	c.4241G>C	p.Cys1414Ser	Missense	-	-	-	Penetrance is less than 60% in this family. Family is classified as isolated ACC.
175	*NOTCH1*	c.5272C>G	p.Arg1758Gly	Missense	0.000004	-	-	This patient also carries a likely pathogenic RBPJ mutation.
16	*NOTCH1*	c.6100T>C	p.Trp2034Arg	Missense	-	-	[2] (Patient 5)	Healthy mother and sister are negative, variants absent in brother with short digits (phenocopy?), no material available from a second healthy brother
		c.6128C>T	p.Ala2043Val	Missense	-	-		
Sporadic probands:								
195	*DLL4*	c.264_266del	p.Phe89delCTT	Inframe deletion	-	-	[3]	No parental material available for screening.
33	*DLL4*	c.799C>A	p.Pro267Thr	Missense	-	[4]	[4] (Family 7)	Heterozygous in unaffected carrier mother, healthy father is negative.
134	*DLL4*	c.1240+5G>C	p.?	Intronic with potential effect on splicing	-	-	-	Classified as VUS due to unavailability of appropriate material to test altered splicing effects.
188	*DOCK6*	c.2104G>A	p.Gly702Ser	Missense	0.002613	[1]	-	-
20	*NOTCH1*	c.1220C>G	p.Pro407Arg	Missense	-	[5]	[5] (Family 7)	Heterozygous in unaffected carrier mother, healthy father is negative.
136	*NOTCH1*	c.1582G>A	p.Asp528Asn	Missense	0.000008	-	-	-
29	*NOTCH1*	c.5218G>T	p.Ala1740Ser	Missense	-	[5]	[5] (Family 9)	No parental material available for screening.
86	*NOTCH1*	c.5452C>G	p.Leu1818Val	Missense	-	-	[6]	No parental material available for screening. This variant has been reported in ClinVar as VUS for an unspecified condition.
194	*NOTCH1*	c.6788G>A	p.Arg2263Gln	Missense	0.000351	-	-	No parental material available for screening. This variant has been reported in ClinVar to be present in a reference population.

All VUS are present in a heterozygous state. MAF, Minor Allele Frequency; VUS, variant of uncertain significance.

[a] GenBank reference sequence and version number for *DLL4*: NM_019074.3; *DOCK6*: NM_020812.3; *NOTCH1*: NM_017617.4; *RBPJ*: NM_005349.3; numbering is from +1 as A of the ATG initiation codon.

[b] gnomAD (http://gnomad.broadinstitute.org/) version r2.0.2 was used.

[c] This column refers to medical case reports in which clinical features observed in specific families are described

Table S4. **Previously published mutations in established AOS genes.**

Gene	Nucleotide change [a]	Amino acid change	Reference
ARHGAP31	c.2047C>T	p.Gln683*	[7]
	c.2063_2064_insTT	p.Ser689*	[8]
	c.3260delA	p.Lys1087Serfs*4	[7]
DOCK6	c.484G>T	p.Glu162*	[1]
	c.788T>A	p.Val263Asp	[1]
	c.1245dupT	p.Asp416*	[9]
	c.1296_1297delinsT	p.Gln434Argfs*21	[1]
	c.1362_1365delAACT	p.Thr455Serfs*24	[1,9]
	c.1902_1905delGTTC	p.Phe635Profs*32	[1]
	c.2520dupT	p.Arg841Serfs*6	[1,10]
	c.3047T>C	p.Leu1016Pro	[1]
	c.3154G>A	p.Glu1052Lys	[1]
	c.3190_3191delCT	p.Leu1064Valfs*60	[11]
	c.4106+5G>T	p.?	[1]
	c.4107-1G>C	p.Thr1370Metfs*19	[10]
	c.4480G>T	p.Glu1494*	[11]
	c.4491+1G>A	p.?	[1]
	c.4786C>T	p.Arg1596Trp	[1]
	c.5235+205_6102-15delinsCATGGGGCTG	p.?	[1]
	c.5939+2T>C	p.?	[1]
DLL4	c.361G>C	p.Ala121Pro	[4]
	c.556C>T	p.Arg186Cys	[4]
	c.572G>A	p.Arg191His	[12]
	c.583T>C	p.Phe195Leu	[4]
	c.799C>A	p.Pro267Tyr	[4]
	c.1168T>C	p.Cys390Arg	[4]
	c.1169G>A	p.Cys390Tyr	[4]
	c.1365C>G	p.Cys455Trp	[4]
	c.1660C>T	p.Gln554*	[4]
	c.1672C>T	p.Arg558*	[4]
EOGT	c.620G>C	p.Trp207Ser	[10]
	c.1074delA	p.Gly359Aspfs*28	[10,13]
	c.1130G>A	p.Arg377Gln	[10]
NOTCH1	Chr9:139439620-139524480del	p.?	[14]
	c.743-1G>T	p.?	[14]
	c.1220C>G	p.Pro407Arg	[5]
	c.1285T>C	p.Cys429Arg	[14]
	c.1343G>A	p.Arg448Gln	[5]
	c.1345T>C	p.Cys449Arg	[5]
	c.1367G>A	p.Cys456Tyr	[5]
	c.1649dupA	p.Tyr550*	[5]
	c.4120T>C	p.Cys1374Arg	[5]
	c.4487G>A	p.Cys1496Tyr	[14]
	c.4663G>T	p.Glu1555*	[5]
	c.4739dupT	p.Met1580Ilefs*30	[5]
	c.5218G>T	p.Ala1740Ser	[5]
	c.5965G>A	p.Asp1989Asn	[14]
	c.6049_6050delTC	p.Ser2017Thrfs*9	[5]
RBPJ	c.188A>G	p.Glu63Gly	[15]
	c.505A>G	p.Lys169Glu	[15]

[a] GenBank reference sequence and version number for *ARHGAP31*: NM_020754.3; *DLL4*: NM_019074.3; *DOCK6*: NM_020812.3; *EOGT*: NM_001278689.1; *NOTCH1*: NM_017617.4; *RBPJ*: NM_005349.3; numbering is from +1 as A of the ATG initiation codon.

Supplementary references

1. Sukalo M, Tilsen F, Kayserili H, et al. DOCK6 Mutations Are Responsible for a Distinct Autosomal-Recessive Variant of Adams-Oliver Syndrome Associated with Brain and Eye Anomalies. *Hum Mutat.* 2015;36(11):1112.
2. Salih MA, Murshid WR, Al-Salman MM, et al. Moyamoya syndrome as a risk factor for stroke in Saudi children. Novel and usual associations. *Saudi Med J.* 2006;27 Suppl 1:S69-80.
3. Itin PH, Bargetzi MC. Aplasia cutis congenita with precancerous transformation - the first case. Why do these scars never develop invasive tumors? *Eur J Dermatol.* 2000;10(3):181-183.
4. Meester JA, Southgate L, Stittrich AB, et al. Heterozygous Loss-of-Function Mutations in DLL4 Cause Adams-Oliver Syndrome. *Am J Hum Genet.* 2015;97(3):475-482.
5. Southgate L, Sukalo M, Karountzos AS, et al. Haploinsufficiency of the NOTCH1 Receptor as a Cause of Adams-Oliver Syndrome With Variable Cardiac Anomalies. *Circulation Cardiovascular genetics.* 2015;8(4):572-581.
6. Fayol L, Garcia P, Denis D, Philip N, Simeoni U. Adams-Oliver syndrome associated with cutis marmorata telangiectatica congenita and congenital cataract: a case report. *American journal of perinatology.* 2006;23(3):197-200.
7. Southgate L, Machado RD, Snape KM, et al. Gain-of-function mutations of ARHGAP31, a Cdc42/Rac1 GTPase regulator, cause syndromic cutis aplasia and limb anomalies. *Am J Hum Genet.* 2011;88(5):574-585.
8. Isrie M, Wuyts W, Van Esch H, Devriendt K. Isolated terminal limb reduction defects: extending the clinical spectrum of Adams-Oliver syndrome and ARHGAP31 mutations. *Am J Med Genet A.* 2014;164A(6):1576-1579.
9. Shaheen R, Faqeih E, Sunker A, et al. Recessive mutations in DOCK6, encoding the guanidine nucleotide exchange factor DOCK6, lead to abnormal actin cytoskeleton organization and Adams-Oliver syndrome. *Am J Hum Genet.* 2011;89(2):328-333.
10. Shaheen R, Aglan M, Keppler-Noreuil K, et al. Mutations in EOGT confirm the genetic heterogeneity of autosomal-recessive Adams-Oliver syndrome. *Am J Hum Genet.* 2013;92(4):598-604.
11. Lehman A, Stittrich AB, Glusman G, et al. Diffuse angiopathy in Adams-Oliver syndrome associated with truncating DOCK6 mutations. *Am J Med Genet A.* 2014;164A(10):2656-2662.
12. Nagasaka M, Taniguchi-Ikeda M, Inagaki H, et al. Novel missense mutation in DLL4 in a Japanese sporadic case of Adams-Oliver syndrome. *J Hum Genet.* 2017.
13. Cohen I, Silberstein E, Perez Y, et al. Autosomal recessive Adams-Oliver syndrome caused by homozygous mutation in EOGT, encoding an EGF domain-specific O-GlcNAc transferase. *Eur J Hum Genet.* 2014;22(3):374-378.
14. Stittrich AB, Lehman A, Bodian DL, et al. Mutations in NOTCH1 cause Adams-Oliver syndrome. *Am J Hum Genet.* 2014;95(3):275-284.
15. Hassed SJ, Wiley GB, Wang S, et al. RBPJ mutations identified in two families affected by Adams-Oliver syndrome. *Am J Hum Genet.* 2012;91(2):391-395.
